# Dynamic Changes of NDVI in the Growing Season of the Tibetan Plateau During the Past 17 Years and Its Response to Climate Change

**DOI:** 10.3390/ijerph16183452

**Published:** 2019-09-17

**Authors:** Xianglin Huang, Tingbin Zhang, Guihua Yi, Dong He, Xiaobing Zhou, Jingji Li, Xiaojuan Bie, Jiaqing Miao

**Affiliations:** 1 College of Earth Science, Chengdu University of Technology, Chengdu 610059, China; huangxianglin@cdut.edu.cn; 2 The Engineering & Technical College of Chengdu University of Technology, Leshan 614000, China; jmiao@mtech.edu; 3 College of Management Science, Chengdu University of Technology, Chengdu 610059, China; 4 Montana Tech of the University of Montana, Butte, MT 59701, USA; 5 International Institute for Earth System Science, Nanjing University, Nanjing 210023, China; DG1927011@smail.nju.edu.cn; 6 Jiangsu Provincial Key Laboratory of Geographic Information Science and Technology, Nanjing University, Nanjing 210023, China; 7 Geophysical Engineering Department, Montana Tech of the University of Montana, Butte, MT 59701, USA; xzhou@mtech.edu; 8 Chengdu University of Technology, College of Environmental and Civil Engineering Institute, Chengdu 610059, China; lijingji2014@cdut.edu.cn; 9 State Environmental Protection Key Laboratory of Synergetic Control and Joint Remediation for Soil & Water Pollution (Chengdu University of Technology), Chengdu 610059, China; 10 College of Tourism and Urban-Rural Planning, Chengdu University of Technology, Chengdu 610059, China; biexiaojuan06@cdut.cn

**Keywords:** NDVI, climate change, Multiple Nested Times Series Analysis (MNTSA), Grey Relational Analysis (GRA), Tibetan Plateau

## Abstract

The fragile alpine vegetation in the Tibetan Plateau (TP) is very sensitive to environmental changes, making TP one of the hotspots for studying the response of vegetation to climate change. Existing studies lack detailed description of the response of vegetation to different climatic factors using the method of multiple nested time series analysis and the method of grey correlation analysis. In this paper, based on the Normalized Difference Vegetation Index (NDVI) of TP in the growing season calculated from the MOD09A1 data product of Moderate-resolution Imaging Spectroradiometer (MODIS), the method of multiple nested time series analysis is adopted to study the variation trends of NDVI in recent 17 years, and the lag time of NDVI to climate change is analyzed using the method of Grey Relational Analysis (GRA). Finally, the characteristics of temporal and spatial differences of NDVI to different climate factors are summarized. The results indicate that: (1) the spatial distribution of NDVI values in the growing season shows a trend of decreasing from east to west, and from north to south, with a change rate of −0.13/10° E and −0.30/10° N, respectively. (2) From 2001 to 2017, the NDVI in the TP shows a slight trend of increase, with a growth rate of 0.01/10a. (3) The lag time of NDVI to air temperature is not obvious, while the NDVI response lags behind cumulative precipitation by zero to one month, relative humidity by two months, and sunshine duration by three months. (4) The effects of different climatic factors on NDVI are significantly different with the increase of the study period.

## 1. Introduction

The Global Climate Change and Terrestrial Ecosystem (GCTE) is one of the core research topics of the International Geosphere Biosphere Programme (IGBP), which has received great attention from the international scientific community as well as the international community in general [1]. Vegetation is a key component of the terrestrial ecosystems, and any change in the terrestrial ecosystems will inevitably lead to fluctuations in vegetation type, quantity or quality. Normalized Difference Vegetation Index (NDVI) is a quantitative parameter that can be used to characterize and reflect the sensitivity of the earth’s surface vegetation coverage and growth status, and it is one of the best indicators for quantitatively characterizing vegetation dynamic changes [2]. At the same time, NDVI is also considered to be an effective indicator of vegetation productivity [3]. Since the 1980s, NDVI has been widely used in studying the impact of climate on vegetation. NDVI dynamics and its correlation with climatic factors have become one of the important research topics of global change [4,5,6]. As the Tibetan Plateau (TP) is an important ecological barrier in Asia [7], in recent decades, it has undergone more severe climate changes than those at the same latitudes [8,9], and the environmental conditions have undergone major changes [10,11,12]. Under this background, NDVI dynamics in TP and their response to climate changes have received extensive attention [8,13,14].

Related studies have found that NDVI changes in the Central Plains of the United States [15], and the Three-River-Source Area in China [16] are closely related to precipitation, while air temperature has greater impact on the NDVI in the northern part of Fennoscandia and parts of North America [17,18]. The relative humidity of semi-arid areas such as Songnen Plain in China has greater impact on NDVI than precipitation [19]. The effect of sunshine duration on NDVI in some parts of Gansu, China is greater than air temperature and precipitation [20]. China is located in the East Asian monsoon region, with complex climate types and rich vegetation types. There are greater differences in vegetation changes and its correlation with hydrothermal factors in different regions. The lag time for vegetation responses to climate factors is also different. Owing to the tight coupling process of surface water heat exchange, warming may have different effects on the Tibetan Plateau (TP) ecosystems. The rise of air temperature can not only reduce the suppression of vegetation activity in the growing season [21,22], but also promote the thawing of surface soil [23,24], and the evaporation increases water loss, which may exacerbate soil water deficit and drought. Many researchers believe that the effects of hydrothermal conditions on the vegetation growth in TP are mutual. Studying the response of NDVI and climatic factors in the vegetation growing season in TP should include the study of hydrothermal factors [25,26,27].

Piao et al. [13] used the correlation analysis method to study the relationship between remotely sensed vegetation quantitative factors and climate change, but the correlation analysis method generally requires each variable to meet the joint normal distribution. The extreme values of the factors in the analysis process will also influence the correlation analysis results [4,28]. Chan et al. [29] and Jin et al. [30] used the Grey Relational Analysis (GRA) method to study the relationship between vegetation index and climatic factors. GRA is considered to be an analysis of the geometric proximity between different discrete sequences within a system. The proximity is described by the grey relational degree, which is regarded as a measure of the similarities of discrete data that can be arranged in sequential order [30,31,32,33]. Differing from the traditional mathematical analysis, GRA provides a simple scheme to analyze the series relationships or the system behavior, even if the input information is less. But the related studies were mainly based on the overall statistical value of the study area or limited sample points. Thus, the results lacked detailed description of vegetation responses to different climate factors in the study area. As for the time period adopted in the research, some scholars [9,34,35] have realized that the linear trend of the overall study period could not fully express the actual dynamic model of vegetation growth change, while the existing studies mainly focus on the change of the whole time period, disregarding continuity of the change process and the variation trend during the time period.

TP is an important ecological barrier for China and even Asia. In recent years, with the increase of global climate change and human activities, the ecosystems of TP have undergone significant changes. At present, correlation analysis method has been taken to study the relationship between NDVI and climate change in TP and analyze the dynamic change of NDVI value in TP and its correlation with climate change in a certain period of time, and few studies focus on the temporal and spatial variation trends of NDVI value in TP and the NDVI value in response to climate change by comprehensively using the methods of Multiple Nested Time Series Analysis (MNTSA) and GRA. In this study, we use the NDVI in TP calculated from the MODIS MOD09A1 data product from 2001 to 2017 to analyze the temporal and spatial variation trends of NDVI values of different vegetation types in the growing season using the MNTSA method. At the same time, combining climatic data such as air temperature, precipitation, relative humidity and sunshine duration of the study area, the lagging response of NDVI to climate factors is analyzed in the growing season of the past 17 years. On the basis of this, combined with the elevation data, the difference of the NDVI value in the growing season and the Grey Relational Degree (GRD) of the climatic factors in the study area at different elevations is analyzed. Together with nested time series, the relationship between vegetation and climatic factors in the growing season is analyzed from the perspective of the influencing degree of NDVI to different climatic factors and the lag time of different climatic factors, so as to promote the deepening and expansion of the research field of vegetation-climate relationship.

## 2. Data and Method

### 2.1. Overview of the Study Area

**Figure 1:**
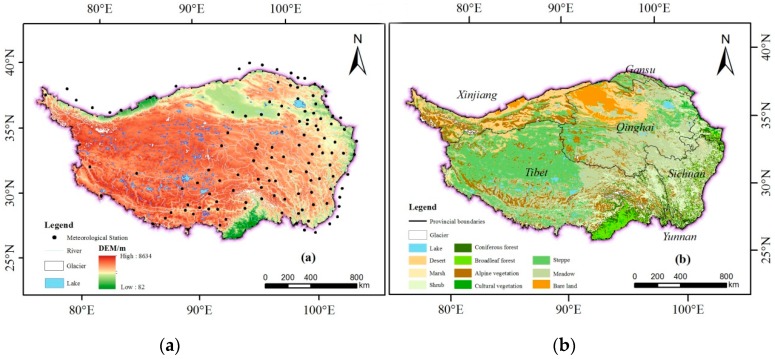
The Digital Elevation Model (DEM), meteorological station distribution (**a**) and the vegetation types (**b**) in TP.

TP has an average elevation of over 4000 m. It is the largest plateau in China with the highest altitude in the world (Figure 1a). It is about 2945 km long from east to west and 1532 km wide from north to south. The total area is about 2.62 × 10^6^ km^2^, accounting for 26.8% of China’s total land area [10].

As the third pole of the earth, TP is at the biological extreme level, and the plateau ecosystem is extremely fragile. It is the launching and sensitive area of global climate change [10,36]. The vegetation in the plateau is extremely sensitive to climate change and TP is one of the hot spots where the global climate change study focuses on [37]. The northwestern TP is cold and arid, while the southeast is warm and humid. The hydrothermal environment in TP is cold at high altitudes, hot and humid at low altitudes. Therefore the vegetation types show a regional differentiation both horizontally and vertically (Figure 1b). The vegetation in the southeastern mountainous area mainly displays a vertical structure, where there are many tropical rain forests, subtropical evergreen forests and cold-temperate coniferous forests from the foothills to the top of the mountain. In the central hinterland, there are mainly alpine meadows, alpine grasslands and alpine grassland, while deserts are widely distributed in the northwest alpine.

### 2.2. Data Sources 

The remote sensing data is derived from the surface reflectance data (MOD09A1) of the Moderate-resolution Imaging Spectroradiometer (MODIS) released by the National Aeronautics and Space Administration (NASA) (https://ladsweb.modaps.eosdis.nasa.gov/). The MOD09A1 product is an 8 d synthetic L3 surface reflectivity product, which contains seven bands. The spatial resolution of the near infrared and red bands is 250 m and for the rest it is 500 m.

The climatic data is selected from the monthly mean air temperature, monthly cumulative precipitation, monthly relative humidity and monthly sunshine duration of the 137 meteorological stations in TP and its neighboring areas from February to September of 2001 to 2017, provided by China Meteorological Science Data Sharing Service Website (http://data.cma.gov.cn).

The vegetation type data is taken from the 1:1,000,000 national vegetation type dataset published by the Resource and Environmental Science Data Center of the Chinese Academy of Sciences (http://www.resdc.cn).

DEM data is taken from Advanced Space borne Thermal Emission and Reflection Radiometer (ASTER) GDEM V2 digital elevation data from Geospatial Data Cloud Platform (http://www.gscloud.cn) with a spatial resolution of 30 m.

The spatial range data of TP comes from the data offered by the Resource and Environmental Science Data Center of the Chinese Academy of Sciences (http://www.resdc.cn).

### 2.3. Data Processing

#### 2.3.1. Data Processing of NDVI

Using the MODIS reprojection tools (MRT), pre-processing such as format conversion, re-projection and image mosaic of the downloaded MOD09A1 data are conducted, and the abnormal value is removed according to the quality control file and the tag file in the MOD09A1 data. NDVI values are calculated by the Band Math of Environment for Visualizing Images (ENVI) software:

(1) $$NDVI=\frac{NIR-R}{NIR+R}$$

In the formula, NIR is the spectral reflectance of the near-infrared band; *R* is the reflectance of the infrared band. The NIR and R from MOD09A1 data correspond to the reflectance of the Band 2 and Band 1, respectively.

**Figure 2:**
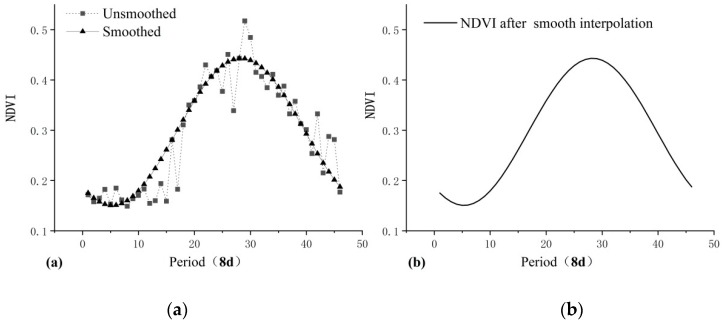
The previous NDVI time series data (**a**) and HANTS processing data (**b**).

The study applies Harmonic Analysis of Time Series (HANTS) to carry out the smoothing process of 8d NDVI data in TP (Figure 2) [38]. The NDVI data processed by HANTS fully reflects the growth curve of vegetation [39,40]. Next, using the method of Maximum Value Composite (MVC), monthly NDVI in the growing season (May-September) are calculated. Subsequently, the effects of bare soil and sparse vegetation areas are eliminated based on the following rules: (1) the annual mean NDVI of the growing season is greater than 0.1; (2) the maximum annual NDVI is greater than 0.15; (3) the maximum annual NDVI should occur from July to September [41,42]. Finally, the mean NDVI from May to September of the year 2001–2017, which meet the pixel requirements above, will be used as the annual mean value of NDVI in the growing season.

#### 2.3.2. Spatial Interpolation of Climatic Data

Distortion of climatic data may occur due to changes in the observation instruments and the surrounding environment of the observation spots. For data from meteorological stations, it is necessary to conduct homogeneity test to eliminate the unavailable data; for some missing data of the meteorological stations, linear regression method is used for interpolation and supplementation [43]. In the existing studies, Thin Plate Spline and Kriging are commonly used for air temperature interpolation [44], Kriging and Thin Plate Spline for precipitation interpolation [45], Kriging and Inverse Distance Weighting (IDW) for relative humidity interpolation [46], and Kriging, Cokriging and other methods for sunshine duration spatial interpolation [47]. In this paper, as the accuracy of station interpolation data is verified, we finally decided to select IDW for relative humidity interpolation, Kriging for precipitation and sunshine duration interpolation. Because air temperature has a certain altitude sensitivity [48], the spatial interpolation of air temperature is carried out by using DEM as the covariate with the interpolation method of the partial thin plate smoothing splines method.

### 2.4. Study Method

#### 2.4.1. Trend Analysis Method

Based on the analysis of the variation trend of NDVI in the growing season in TP during 2001–2017 with the method of linear regression, the formula is:

(2) $$\theta_{slope}=\frac{n\times \sum_{i=1}^{n}\left(i\times {NDVI}_{i}\right)-\sum_{i=1}^{n}i\sum_{i=1}^{n}{NDVI}_{i}}{n\times \sum_{i=1}^{n}i^{2}-{\left(\sum_{i=1}^{n}i\right)}^{2}}$$

where *n* represents a time series, *n* = 17; *NDVI_i_* represents the NDVI value of the year *i*; *θ_slope_* is the inter-annual variation slope of a pixel NDVI; positive value of *θ_slope_* indicates that the NDVI shows an increasing trend, and vice versa. According to the results of the significance test, the trend is divided into three levels: extremely significant (*p* < 0.01), significant (*p* < 0.05), and insignificant (*p* ≥ 0.05) [9].

#### 2.4.2. Grey Relational Analysis

Multi-variate statistical analysis often uses correlation analysis and regression analysis. The multiple regression analysis method requires samples to obey a typical probability distribution, which has a large amount of calculation and a complicated calculation process. Correlation analysis method generally requires that each variable meet the joint normal distribution, and in the analysis process, the extreme value of the factor will have a greater impact on the relevant analysis results, and even lead to the distortion of the essential association among the factors [49]. GRA is an analysis method that describes the strength, size, and order of relationships between factors using a grey relational order. The GRA has the characteristics of not being restricted by the type of samples and the probability distribution, known as an effective method to study an uncertain issue with little data and information. The basic idea of GRA is based on the geometric similarity of curves. The curves of two series are more similar, the series are more relevant, and vice versa [31]. It overcomes the shortcomings of traditional mathematical statistical analysis methods to some extent. Wong et al. [32] and Ip et al. [33] utilized GRA to analyse change-points in hydrological time series and water environment quality, and found that GRA should be expected to be applicable in other disciplines, too. But the related studies were mainly based on the overall statistical value of the study area or limited sample points, thus the results lacked detailed description of the distribution of GRD in the study area. The basic steps and related formulas of GRA are as follows:

We first determine the reference factor sequence and the comparative factor sequence. The dependent variable constitutes the reference sequence *X*_0_, and the independent variable constitutes a comparative sequence *X_i_* (*i* = 1, 2, ..., m), and *X*_0_ and *X_i_* are called variable sequences. In this study, the NDVI value in the growth season in 2001–2017 is used as the reference factor sequence (*X*_0_), and the comparative factor sequence (*X_i_*) consists of four climatic factors: air temperature (*X*_1_), precipitation (*X*_2_), relative humidity(*X*_3_), and sunshine duration (*X*_4_) in 2001–2017. 

The initial value method is used to perform the dimensionless processing of sequence variables. The initial value pixel of each sequence can be expressed as:

(3) $$X_{i}^{\prime }=\frac{X_{i}}{x_{i}(1)}=(x_{i}^{\prime }(1),x_{i}^{\prime }(2),\cdots ,x_{i}^{\prime }(n)),i=0,1,2,\cdots ,m$$

We then calculate the difference sequence, the maximum difference and the minimum difference. The absolute value of the difference between *x_0′_* and *x_i_^′^* corresponding point *k* (*k* = 1, 2, ..., *n* represents the number of observation objects) constitutes a difference sequence. In this study, *x_0′_*(*k*) is the value of the NDVI pixel processed by the initial value method in the *k* year and *x_i_′*(*k*) is the value of climatic factors pixel processed by the initial value method in the *k* year.

The difference sequence can be expressed as:

(4) $$\Delta_{i}(k)=\left|x_{0}^{\prime }(k)-x_{i}^{\prime }(k)\right|\;\Delta_{i}=(\Delta_{i}(1),\Delta_{i}(2),\ldots ,\Delta_{i}(n)),i=1,2,\ldots ,m$$

The maximum difference and minimum difference between the two poles are expressed as:

(5) $$M=\underset{i}{\max}\underset{k}{\max}\Delta_{i}(k),m=\underset{i}{\min}\underset{k}{\min}\Delta_{i}(k)$$

To calculate the grey relational coefficient, the relational coefficient can be expressed as:

(6) $$r_{0i}(k)=\frac{m+\xi M}{\Delta_{i}(k)+\xi M},\xi \in (0,1),k=1,2,\ldots ,n;i=1,2,\ldots ,m$$

To calculate the Grey Relational Degree (GRD), the formula for GRD is:

(7) $$r_{0i}(k)=\frac{1}{n}\sum_{k=1}^{n}r_{0i}(k),i=1,2,\ldots ,m$$

where *r_0i_* is the GRD of *x_i_* for *x*_0_ under the condition that it meets the variation factor *k* and the grey-scale resolution coefficient *ξ*. *ξ* ∈ (0,1) is the distinguishing coefficient, usually, *ξ* = 0.5. The GRD is a measure of the influence of the comparative factor sequence on the reference factor sequence. When the value is closer to 1, the comparative factor sequence has a more significant influence on the reference factor sequence. The GRD can be used as a comparison of the influence of the comparative factor sequence on the reference factor sequence.

#### 2.4.3. Analysis of Lag Time

Vegetation has certain adaptability to climate change. Only when climate change reaches a certain level, can vegetation change take place, that is, the response of vegetation to climate change has a certain time lag. The NDVI sequence of the growing season in the study area from 2001 to 2017 (May–September) and the monthly mean air temperature sequence of the growing season are taken as two groups of variables for calculating the GRD between NDVI and monthly mean air temperature. Similarly, to calculate the GRD of NDVI sequence of the growing season and the monthly mean air temperature (April–August, March–August, and February–June), we use monthly cumulative precipitation (May–September, April–August, March–July and February–June), monthly mean relative humidity (May–September, April–August, March–July, and February–June) and monthly cumulative sunshine duration (May–September, April–August, March–July, and February–June), respectively. By comparing NDVI in the growing season with the GRD of the same comparative factor at different moments, the lag time of the NDVI to the climatic factors is discussed in the growing season.

#### 2.4.4. Multiple Nested Times Series Analysis

In this paper, the method of MNTSA proposed by Du et al. [9] is used to discuss the response process of NDVI changes in TP to climate change. MNTSA is a method with a fixed starting year and a gradual increase of the ending year. This paper selects the growth period from 7 periods: 2001–2011, 2001–2012, ..., 2001–2017 to calculate the NDVI variation trends and their responses to climatic factors.

MNTSA may face the Modifiable Temporal Unit Problem (MTUP) in statistics, but studies have shown that the MTUP effect can be ignored when multiple nested time series method is applied to calculate NDVI variation trends and their responses to climatic factors [50,51,52]. At the same time, the method is prominent in reflecting the dynamic process of NDVI, the persistence and stability of the variation trend. Long-term time series analysis of NDVI based on multiple nested periods helps to understand the persistence of NDVI variation process and variation trends, and it can reflect the elasticity of the vegetation system and the resistance to external disturbances to some extent.

## 3. Results and Analysis

### 3.1. Interannual Variation and Spatial Distribution of NDVI in the Growing Season

#### 3.1.1. Interannual Variation of NDVI

**Figure 3:**
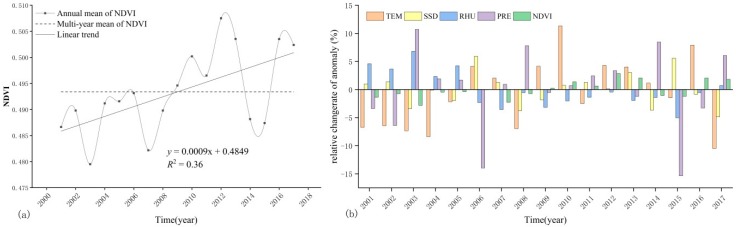
Dynamic changes of NDVI (**a**) and climate factors (**b**) in TP during 2001–2017 (TEM-Air temperature, SSD-sunshine duration, RHU-Relative humidity, PRE-Precipitation).

According to the annual mean NDVI in the growing season of TP from 2001 to 2017, the interannual variation of NDVI during the 17-year period is obtained (Figure 3a). In the study periods, the mean NDVI in the growing season shows a upward trend. The annual mean NDVI of the growing season is 0.49 with a growth rate of 0.01/10a (R^2^ = 0.36, *p* = 0.01). The mean NDVI of 2001–2008, 2014 and 2015 is lower than the multi-year mean level. The mean NDVI of 2009–2013, 2016 and 2017 is higher than the multi-year mean level. The lowest value of NDVI (0.48) appears in 2003, the highest value (0.51) appears in 2012. The mean NDVI in the growing season of TP shows small peaks in 2010, 2012 and 2017 respectively. In 2003, 2007, 2014 and 2015, there are obvious low values, which may attribute to the hydrothermal environment of the year. According to the statistics of climatic data (Figure 3b), the positive anomaly of mean air temperature in 2010 is 11.33%, the relative humidity shows negative anomaly, the sunshine duration and the precipitation don’t change much, and an increase of air temperature may be more suitable for vegetation growth when other climatic conditions are relatively stable. The NDVI in the growing season reaches its maximum in 2012, which may be related to the increase of air temperature and precipitation in the year. In 2016, only air temperature shows positive anomaly. Although relative humidity changes greatly, the anomaly of precipitation and sunshine duration is small, and the rise of air temperature reduces the suppression of vegetation activity in the growing season. In 2003, there is a decrease of air temperature, a significant decrease of sunshine duration, a significant increase of precipitation and relative humidity. The decrease of air temperature and sunshine duration led to suppression of vegetation growth. The NDVI in the growing season of 2007 is lower than that in 2006, and the air temperature and the sunshine duration of the year shows positive anomaly, while the relative humidity is negative, and the precipitation does not change much. However, the precipitation decreases severely in the previous year, and the percentage of positive anomaly in air temperature and sunshine duration are 4.13% and 5.94%, respectively. This warm-drying climate change is not conducive to vegetation growth and may affect the NDVI of the next year’s vegetation growing season. The NDVI values in the growing season of the study area in 2014 and 2015 are both low, but the causes of this result are different. The hydrothermal conditions in 2014 shows that precipitation increased sharply and sunshine duration reduce significantly when air temperature and relative humidity do not change much. This phenomenon leads to suppression of vegetation growth. In 2015, the NDVI value in the growing season of the study area is relatively low. The percentage of positive anomaly of sunshine duration is 5.59%, and relative humidity and precipitation display negative anomaly, 5.03% and 15.35%, respectively. The hydrothermal combination of drastically reduced precipitation as well as relative humidity, and the significant increase of sunshine duration, are likely to result in drought and a decrease in the NDVI value.

#### 3.1.2. Multi-Year Mean Spatial Distribution of NDVI

**Figure 4:**
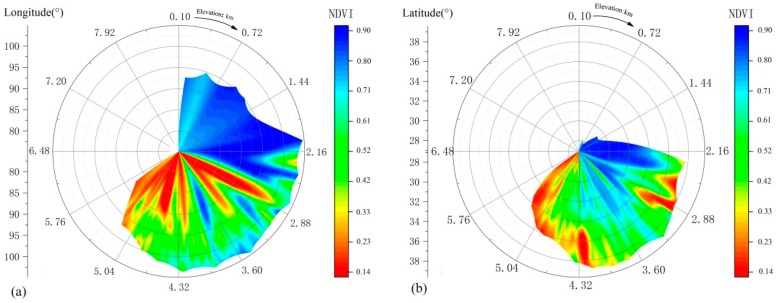
Spatial distribution of multi-year mean of NDVI in the growing season in TP from 2001 to 2017. (**a**) Statistics based on longitude and altitude, (**b**) Statistics based on latitude and altitude.

The relative difference in height of TP is large, and the diverse climate types and the vegetation’s strong sensitivity to climate result in uneven spatial distribution of the ecological patterns. Overall, the NDVI distribution in the growing season shows a trend of decreasing from east to west, with a change rate of −0.13/10° E (Figure 4a), and gradually decreasing from north to south, with a change rate of −0.30/10° N (Figure 4b). 

The statistical results show that the high-value (0.4–1) areas of NDVI in the vegetation growing season have an average elevation of about 4100 m, mainly distributed in the central and western parts of Sichuan Province, southwestern Gansu Province, the western and southwestern parts of the Qinghai Province, and the eastern part of the Tibet Autonomous Region from 26° to 39° N and from 86° to 104° E. In this region, there are mainly cultivated vegetation, coniferous forests, broad-leaved forests and meadows. The low-value (<0.4) areas have an average elevation of about 4600 m, mainly distributed in the central part of the Qinghai Province, most parts of the Tibet Autonomous Region, northwestern part of the Xinjiang Uygur Autonomous Region, northwestern Sichuan Province, northern Yunnan Province and the northwestern part of Gansu Province from 27° to 39° N and from 76° to 106° E, where the vegetation types are mainly shrubs and alpine vegetation.

### 3.2. Spatial Variation Characteristics of NDVI in the Growing Season of the Past 17 Years

**Figure 5:**
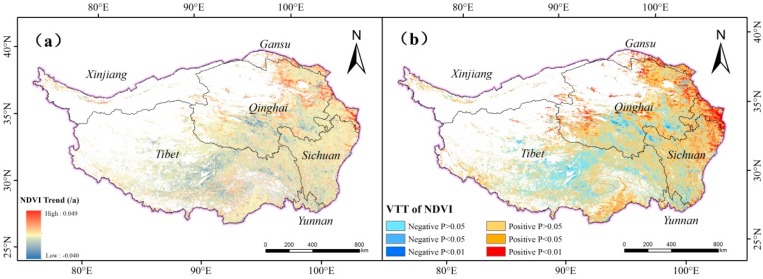
NDVI trend (**a**) and Variation Trend Types (VTTs) of NDVI (**b**) in TP from 2001 to 2017.

With the method of trend analysis, the variation trend of NDVI in the growing season of the study area in the 17 years is obtained (Figure 5a). Based on the trend analysis of the NDVI in the study area, the variation trend of NDVI in the growing season of 2001–2017 is divided into six types according to the significance test results: extremely significant reduction (*θ_slope_* < 0, *p* < 0.01), significant reduction (*θ_slope_* < 0, *p* < 0.05), not significant reduction (*θ_slope_* < 0, *p* > 0.05), not significant increase (*θ_slope_* > 0, *p* > 0.05), significant increase (*θ_slope_* > 0, *p* < 0.05) and extremely significant increase (*θ_slope_* > 0, *p* < 0.01) (Figure 5b). In general, the NDVI values of the growing season in the study area shows an increasing trend from 2001 to 2017. The significance test of the NDVI trend analysis in the study area indicates that the ratio of area showing an increasing trend of NDVI in the growing season of the study area is 68.11%. Areas with significant increase (15.40%) are mainly distributed in the Gansu, eastern Sichuan, central and western Qinghai, and southeastern Tibet while areas with extremely significant increase (7.31%) are mainly located in the central and eastern parts of the study area. The ratio of area showing decreasing trend of NDVI in the growing season is 31.89% (the areas with extremely significant reduction and significant reduction accounting for 0.89% and 2.50% of the total area respectively). Areas with significant reduction, extremely significant reduction are mainly distributed in the central Qinghai and central Tibet.

The study area is divided into four altitude ranges: low altitude area (<1000 m), middle altitude area (1000–3500 m), high altitude area (3500–5000 m) and extremely high altitude area (>5000 m) [53]. The NDVI interannual variation trend shows significant differences at different elevation ranges (Table 1). The areas with increasing trend of NDVI at all altitude ranges are larger than those with decreasing trend. Among low altitude areas (81.22% vs. 18.78%), medium altitude areas (80.81% vs. 19.19%), high altitude areas (65.8% vs. 34.2 %), extremely high altitude areas (62.7% vs. 37.3%), the middle altitude areas cover the largest area where NDVI value shows extremely significant increase (20.03%) and significant increase (33.14%), while extremely high altitude areas cover the smallest area with extremely significant increase (3.54%) and significant increase (19.32%). It shows that the improvement of vegetation in the middle and low-altitude areas is better than that of high-altitude and extremely high-altitude area in 2001–2017. The proportion of area where NDVI value of vegetation shows extremely significant increase and extremely significant reduction in the mid-altitude area is the largest among the four elevation ranges, probably due to human factors in the mid-altitude area.

### 3.3. Temporal Variation Trend of NDVI in the Growing Season

**Figure 6:**
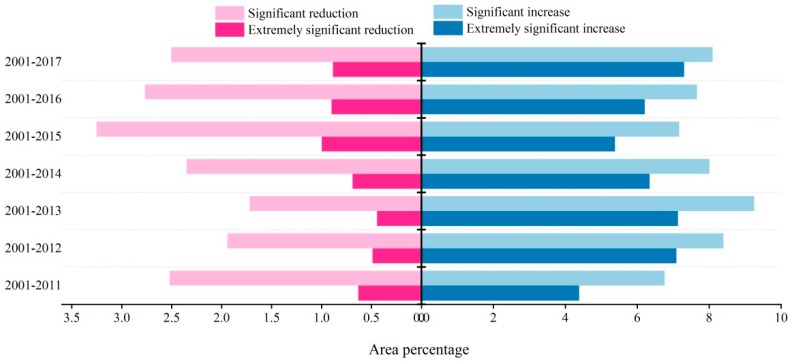
The proportion (%) of different NDVI variation trend in seven time periods.

From the periods of 2001–2011, 2001–2012, ..., 2001–2017, the proportion of area showing different NDVI variation trends in the growing season of TP tells that areas where NDVI values showing an increasing trend do not display a visible trend as the study period prolongs. In terms of all periods in the growing season, areas with increasing trend of NDVI values are larger than those with decreasing trend, and the areas with extremely significant increasing trend are also larger than those with significant reduction trend. Areas with extremely significant reduction trend of NDVI value in the growing season show an obvious trend, and the growth rate is greater than that of the extremely significant increase area (Figure 6). It indicates that the NDVI value in the growing season of TP has mainly shown an increasing trend in the past 17 years. Under the background of the overall increase of NDVI value, the area with decreasing NDVI is gradually expanding.

**Figure 7:**
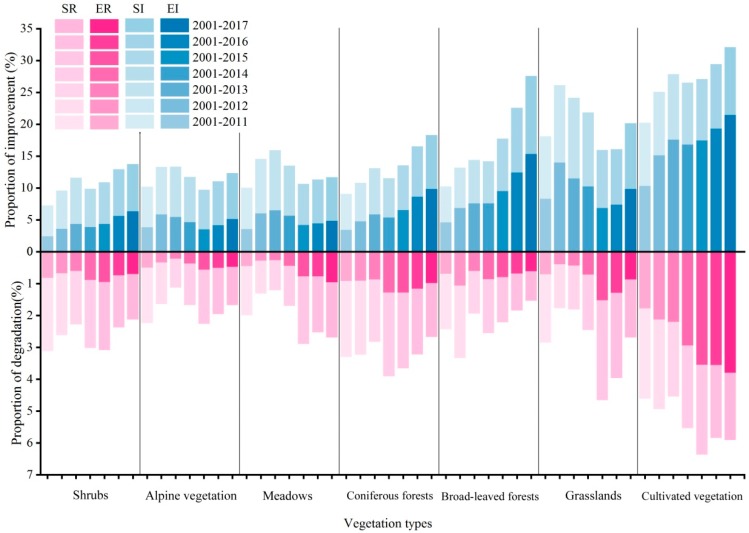
The proportion (%) of different NDVI variation trends of different vegetation types in seven time periods (SR - Significant reduction, ER - Extremely significant reduction, SI—Significant increase, EI—Extremely significant increase).

In terms of vegetation types (Figure 7), the overall NDVI value of each vegetation type increases by more than 50%, but the areas with increasing NDVI in meadows and grasslands show a shrinking trend, and areas with a significant increase show a shrinking trend, while areas with significant reduction display a trend of expansion. The areas with significant increase of NDVI value of cultivated vegetation show a trend of expansion, so does the area with significant reduction. The area with increasing NDVI value of the forest area continues to increase. The above studies show that the variation trend of some NDVI will be covered by the overall change in the background of an overall increase of NDVI value. Previous studies have shown that climate warming has greatly increased the evaporation in TP. Therefore, the increase of air temperature can promote vegetation growth, on the other hand, it may also suppress the growth of some vegetation, and human disturbance such as overgrazing will lead to grassland degradation [26,27]. Therefore, areas with increasing trend and significantly increasing trend of NDVI in meadows and grasslands are reducing, which is opposite to the overall variation trend. It is likely to take place due to the synergy of climate warming and human disturbance. The areas with significantly increasing trend and significant reduction trend of NDVI show a trend of expansion, indicating that there coexists returning farmland to grass or returning farmland to forest and abandoned farming in cultivated vegetation. The areas with significant increasing trend and extremely significant increasing trend of NDVI in forest land (coniferous forest and broad-leaved forest) are continuously increasing, which may result from the protection measures of natural forest resources and returning farmland to forests.

### 3.4. Discussion on the Lag Response of NDVI to Climate Factors in the Growing Season

According to the statistical results of the GRD and the NDVI in the growing season of the study area in 2017 to mean air temperature, cumulative precipitation, mean relative humidity and cumulative sunshine duration in different time sequences (Table 2) show that during the growing season, the NDVI has the highest average GRD with mean air temperature from May to September (0.613), with cumulative precipitation from May to September and from April to August (0.616), with relative humidity from March to July (0.614), and with cumulative sunshine duration from February to June (0.631). It indicates that the NDVI of the growing season in the study area lags behind air temperature not obviously, cumulative precipitation by 0–1 month, relative humidity by 2 months, and sunshine duration by 3 months. That is, the vegetation in the growing season of the study area responds more quickly to air temperature and precipitation, while the response to relative humidity is slower, and the response to sunshine duration is the slowest.

In order to study the dynamic change of NDVI response to climatic factors in the growing season, with the method of multiple nested time series analysis, we divide 2001–2017 into seven periods, 2001–2011, 2001–2012, 2001–2013, …, 2001–2017 and conduct GRA, respectively. The statistical results (Table 3) show that the response of the NDVI to mean air temperature in the growing season of the seven periods from 2001 to 2017 occurs in the current month, but the lag is not obvious, the response time to cumulative precipitation is gradually shortening; in 2001–2011, 2001–2012 and 2001–2013 the response of the NDVI lags behind cumulative precipitation by one month, but after 2014, it basically responded in the current month; the response of the NDVI to relative humidity lags behind by 2 months; the response of the NDVI to sunshine duration lags behind by 3 Months. The response time of NDVI to climatic factors (except cumulative precipitation) in the seven study periods almost remain the same, and the lag response time of NDVI to climate factors has higher stability.

### 3.5. Analysis of NDVI Response to Climatic Factors in Growing Season

**Figure 8:**
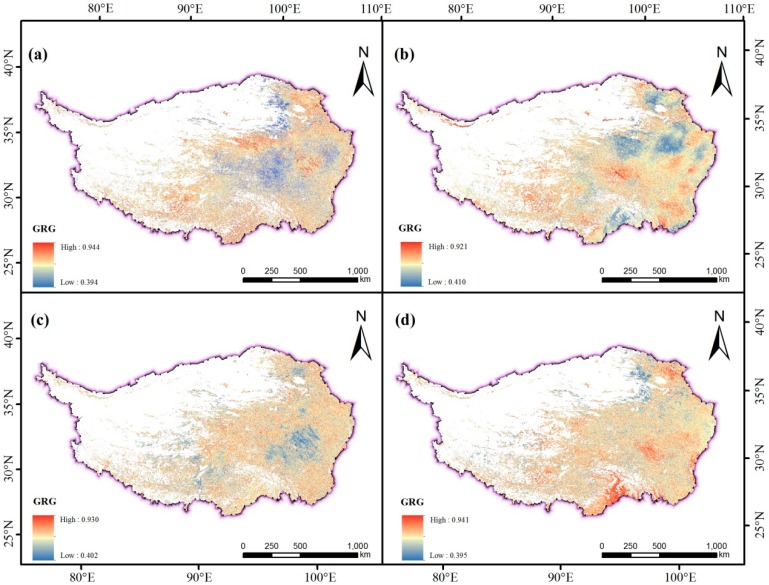
GRD between NDVI and air temperature (**a**), precipitation (**b**), relative humidity (**c**), and sunshine duration (**d**).

Based on the results of the lag response analysis, the total time period (2001–2017) is selected to analyze the response of NDVI to climatic factors. The correlation analysis between NDVI and air temperature in the study area (Figure 8a) shows that the GRD between NDVI and air temperature is between 0.394 and 0.944, with an average of 0.613. The high-value (>0.613) area is mainly distributed in the study area at the junction of the Sichuan and Qinghai provinces, the central and southern part of Qinghai and the central and southern part of Tibet. The vegetation types are mainly meadows, grasslands, coniferous forests, broad-leaved forests, alpine vegetation and so on. The low-value (<0.613) area is mainly distributed at the junction of Tibet, Sichuan and Qinghai, and the vegetation types are mainly meadows and shrubs.

**Figure 9:**
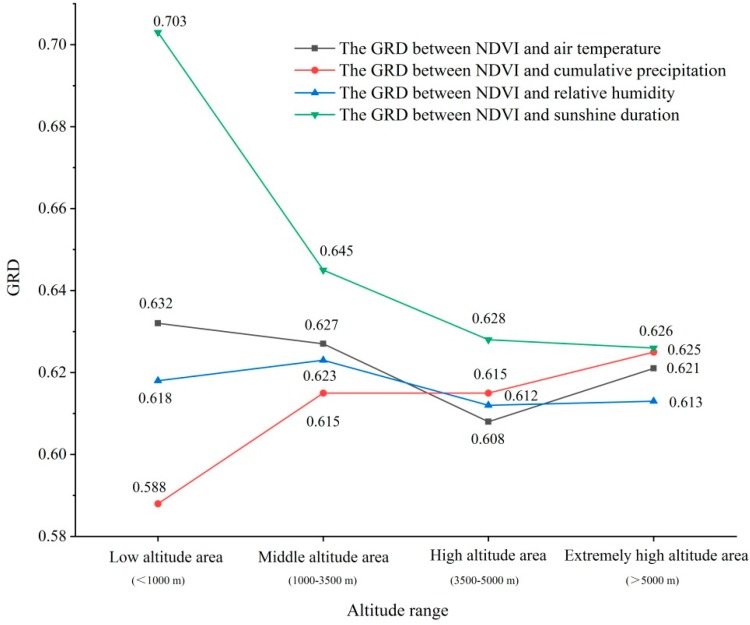
Variation of GRD between NDVI and air temperature, precipitation, relative humidity and sunshine duration at different elevations.

The GRD of the low altitude area is 0.632, the middle altitude 0.627, the high altitude 0.608, and the extremely high altitude 0.621 (Figure 9). On the whole, with the increase of altitude, the GRD between NDVI and mean air temperature shows a downward trend. When the altitude ranges from middle to high, the GRD drops sharply to 0.608, but roses to 0.621 at extremely high altitude, indicating that the correlation between NDVI and air temperature is higher at extremely high-altitude, mid-altitude and low-altitude areas, and the correlation between NDVI and air temperature reduces due to complex hydrothermal conditions and extremely harsh natural environment at high altitude.

On the time scale of 17 years, the GRD between NDVI and cumulative precipitation in TP is between 0.410 and 0.921 (Figure 8b), with an average of 0.616. Among them, the NDVI values of grasslands, meadows, coniferous forests and shrubs, which are distributed at the junction of the Tibet and eastern Qinghai Province, the central part of Qinghai Province and the northern part of Yunnan Province, have a relatively high correlation with the cumulative precipitation. The NDVI values of meadows, broad-leaved forests and grasslands distributed in large areas of eastern Qinghai Province and southeastern part of Tibet are less correlated with cumulative precipitation. The GRD of low-altitude area is 0.588, the mid-altitude 0.615, the high-altitude 0.615, and the extremely high-altitude 0.625 (Figure 9). With the increase of altitude, the NDVI is affected by accumulative precipitation, and the low-altitude area is basically located in the southeastern part of TP, where rivers are densely covered and abundant precipitation causes the correlation between NDVI and precipitation to be relatively low.

The correlation analysis between NDVI and relative humidity in the study area shows that, the GRDs between NDVI and relative humidity ranges from 0.402 to 0.93 with an average of 0.614 (Figure 8c). The high-value area (>0.614) is evenly distributed throughout the study area. The main vegetation types are grassland, meadow, coniferous forest, and alpine vegetation. The low-value (<0.614) area is mainly distributed in the eastern part of the study area, northwestern Sichuan Province, central Tibet and northeastern Qinghai Province. The vegetation types include meadows, grasslands, broad-leaved forests and shrubs. The correlation between NDVI and relative humidity at different altitudes ranks in descending order: middle-altitude area (0.623), low-altitude area (0.618), extremely high-altitude area (0.613) and high-altitude area (0.612) (Figure 9). In the middle and low altitude areas, the correlation between NDVI value and relative humidity is the highest in the growing season, while the correlation between NDVI and relative humidity is relatively low due to the dry climate in the high-altitude areas and extremely high-altitude areas.

The correlation between NDVI and sunshine duration in different areas is significantly different (Figure 8d). The grey correlation between NDVI and cumulative sunshine duration ranges from 0.395 to 0.941, with an average of 0.631, and the NDVI values of meadows, shrubs and broad-leaved forests are highly correlated with the duration of sunshine in southeastern Tibet, eastern Tibet and northeastern part of the study area. The high-value (>0.631) areas are more evenly distributed in the study area, especially concentrated in the southeastern part of Tibet, eastern Tibet and the northeastern part of the study area. The vegetation types are mainly meadows, shrubs and broad-leaved forests. The low-value (<0.631) areas are distributed in the study area and is concentrated in the northeastern part of the Tibetan Plateau, where the vegetation types are mainly grassland and meadow. The correlation between NDVI of the growing season and sunshine duration in the low-altitude area is 0.703, the mid-altitude area 0.645, the high-altitude area 0.628, and the extremely high-altitude area 0.626 (Figure 9). It indicates that in the low-altitude area of the study area, most of the areas are affected by the duration of sunshine, and the extent of effect is much higher than that of the middle, high and extremely high altitude area. With the increase of altitude, the influence of sunshine duration on vegetation gradually declines. The reason may be that the low-altitude area is located in the southeastern part of the study area, where clouds are thick, and the southeastern part of TP receives less solar radiation than other parts of the plateau. Therefore, vegetation in the high southeastern part has high sensitivity of the abnormal sunshine duration. The correlation between NDVI and sunshine duration at high altitude and extremely high altitude does not reduce with the dramatic change of altitude.

The GRD between NDVI and climatic factors in the growing season of TP is significantly different. The GRD between NDVI and air temperature is the highest (0.632) in the low-altitude area, and the lowest (0.608) in the high-altitude area; the GRD with the sunshine duration is the highest (0.703) in the low-altitude area, and the lowest (0.626) in the extremely high-altitude area; the GRD with relative humidity is the highest (0.623) in the middle-altitude area and the lowest (0.612) in the high-altitude area; the GRD with precipitation is the highest (0.625) in the extremely high-altitude area and the lowest (0.588) in the low-altitude area. The cumulative sunshine duration of the study area is more dominant than the other climatic factors and the NDVI of the growing season has the closest relation to the duration of sunshine. In the middle and low altitude areas, the response of NDVI to air temperature and relative humidity is much higher than that of precipitation. The correlation between sunshine duration and cumulative precipitation in the middle and low altitude areas is generally negative, and in the area where the correlation with sunshine duration is high, the correlation with cumulative precipitation is low, but the difference of correlations between the remaining climatic factors are more obvious. In high-altitude areas, the response of NDVI to precipitation is much higher than that of air temperature and relative humidity. The correlation between sunshine duration, precipitation, air temperature and relative humidity is quite different, but the correlation between sunshine duration and air temperature in eastern Haixi County reflects certain consistency. At extremely high-altitude area, the response of NDVI to precipitation is much higher than that of air temperature, and the response to air temperature is much higher than that of relative humidity. In extremely high-altitude area, the natural environment is extremely bad, and the response of NDVI to climate factors is quite different. Since the amount of thick clouds can easily reduce sunshine hours [53], the correlation between NDVI and sunshine duration is often low in the area where NDVI is highly correlated with relative humidity and cumulative precipitation, and the correlation between NDVI and sunshine duration in the region where NDVI is associated with relative humidity and cumulative precipitation is often higher.

## 4. Discussion

Piao et al. [54] analyzed the correlation between NDVI and climatic variables in temperate grasslands of China from 1982 to 1999, and found that NDVI in China’s temperate grasslands has certain degree of lag to climate change. Wang et al. [15] studied the response of NDVI to climatic factors in Kansas from 1989 to 1997 and found that different vegetation types responded to air temperature and precipitation in different ways, and had different lag times. Ding et al. [7] utilized the NDVI data of NOAA/AVHRR satellites from 1982 to 2000 to study the response and spatial characteristics of vegetation cover in TP to hydrothermal conditions during the year, and considered that in TP, except for forests, correlation between NDVI of all vegetation types and precipitation is higher than that of air temperature. The NDVI of TP has a lag effect on air temperature and precipitation, and there is a spatial difference in the lagging level, ranging from 10 days to 90 days. Du et al. [55] used multiple nested time series to analyze the variation trend of NDVI in the vegetation growing season from 1982 to 2012 and the response relationship with climatic factors. They found that the area where vegetation activity in TP increased or decreased significantly would continue to expand, but the rate of expansion in areas with significant reduction is significantly higher than that of the significantly increased area.

Regarding the lag time of NDVI to climatic factors in the growing season, based on the data of MOD09A1 8d, the response of NDVI to climatic factors in TP shows that the response to air temperature is not obvious, but lags behind precipitation by 1 month, relative humidity by 1–2 months, and sunshine duration by 2–3 months. This is different from what Ding et al. [42] has found that the NDVI of TP lags behind air temperature and precipitation by 10 days to 90 days, largely due to: (1) in the overall context of global warming, the hydrothermal environment of TP has undergone major changes recently [9,56]. (2) In the past studies on vegetation of TP, the NOAA/AVHRR NDVI data was used, but the AVHRR sensor has a wide band in the visible and near-infrared bands, and contains several strong water vapor absorption bands, which reduces the reliability of the calculated NDVI. Compared with AVHRR, the visible spectrum of the MODIS sensor increases the sensitivity to chlorophyll, while the near-infrared band eliminates the interference of atmospheric water vapor [57]. (3) Past studies conducted lag analysis on single-month NDVI and climatic data, ignoring the cumulative effect of climatic factors [8,55]. In this study, several months of climatic data have been used. Based on the NDVI data of the growing season (May–September), we calculated the GRD of mean air temperature, cumulative precipitation, relative humidity and sunshine duration for current month (May–September), the previous one month (April–August), the previous two month (March–July), and the previous three month (February–June). On the basis of the calculated GRDs, the response time of NDVI to climatic factors in the growing season of TP is obtained.

This paper discusses the response of NDVI variation in the vegetation growing season of TP to climatic factors (air temperature, precipitation, relative humidity and sunshine duration). In addition, there is a significant correlation between vegetation growth and extreme weather. Relevant research indicates that the minimum air temperature and water vapor pressure will have an important impact on the NDVI variation in TP during the year, while the potential evapotranspiration and wind speed will have an important impact on interannual variation of NDVI [58]. The NDVI variation is not only closely related to climate change, but also closely related to human activities and other factors [59]. Before the 21st century, especially in the 1980s and 1990s, due to excessive grazing, deforestation or farmland, the destruction of surface vegetation was severe, resulting in the continuous deterioration of the ecological environment in TP, serious soil erosion on the plateau, and threatened biodiversity [60,61]. Since the beginning of the 21st century, China has implemented a series of ecological restoration projects in TP [62]. The ecological environment of the study area has been effectively protected, and the improvement of the ecological environment of forest land is particularly obvious [63]. In semi-arid areas, human management of vegetation ecological zones has become the main driving force for vegetation change [7,64]. Similar to many studies, this study found that the NDVI values of grasslands and meadows in the central and eastern TP continued to rise during 2001–2017, and the extensive implementation of ecological restoration measures for planting trees, grass grazing, and sand control played an important role in the process. Owing to the superposition of human factors, the sensitivity of vegetation growth to climatic factors changes, which ultimately leads to differences in the degree and speed of response of NDVI to air temperature, precipitation, relative humidity and sunshine duration.

## 5. Conclusions

Based on the MOD09A1 remote sensing data, vegetation type data, topographic data and four climatic data sets, this paper analyzes the spatial and temporal distribution patterns and trends of NDVI in the growing season of TP from 2001 to 2017. The GRA method is used to study the lag time of NDVI response to climatic factors in TP for 17 years. With the method of multiple nested time series, NDVI trends and dynamic change of NDVI response to climatic factors in seven periods are analyzed, and the main conclusions are as follows:

(1) The mean NDVI in the growing season of TP in 2001–2017 is 0.49, and the spatial difference is significant. The NDVI value of vegetation generally shows a spatial distribution of increase from west to east and from south to north. The change rate from west to east is 0.13/10° E, while the change rate from south to north is 0.30/10° N.
(2) During the period from 2001 to 2017, the NDVI in the growing season of TP shows a slight trend of increase, with a growth rate of +0.01/10a.
(3) The results of the seven periods indicate that the area where overall NDVI value of TP shows an increasing trend is enlarging with the extension of the study period. The area where NDVI value of meadows and grasslands shows a decreasing trend is declining, and the areas with significant increase and extremely significant increase of the NDVI value in forest lands (coniferous forests and broad-leaved forests) are increasing significantly.
(4) The results of lag time show that the response of the NDVI in TP to the four climate factors (air temperature, precipitation, relative humidity, and sunshine duration) varies from 2001 to 2017, and the lag of air temperature is not obvious, and the response lags behind cumulative precipitation by zero to one month, relative humidity by two months, and sunshine duration by three months. The results of the seven periods show that the NDVI of the growing season and the lag time response of air temperature, precipitation, relative humidity and sunshine duration remain basically unchanged at the existing research scale, and the vegetation response time to climatic factors has certain stability.
(5) The correlation between NDVI and climatic factors in different altitudes shows significant differences. The correlation between NDVI and air temperature in the study area is the highest (0.632) in the low-altitude area and the lowest (0.608) in the high-altitude area. The highest correlation (0.625) with precipitation is located in extremely high-altitude area and the lowest correlation (0.588) in the low-altitude area). The highest correlation (0.623) with relative humidity occurs in the low-altitude area, while the area with the lowest correlation (0.612) is located in the extremely high-altitude area. The highest correlation (0.703) with sunshine duration occurs in the low-altitude area, while the area with the lowest correlation (0.626) is located in the extremely high-altitude area. The GRD between NDVI and sunshine duration decreases with the increase of altitudes. The GRD between NDVI and precipitation increases as the altitudes rise. The change of GRD between NDVI and relative humidity is relatively stable in different altitudes. The GRD between NDVI and air temperature fluctuates with the rise of altitudes.

## Figures and Tables

**Table 1:** Statistics of NDVI changes in the TP from 2001 to 2017.

Area Percentage	Low Altitude Area <1000 m	Middle Altitude Area 1000–3500 m	High Altitude Area 3500–5000 m	Extremely High Altitude Area >5000 m
Increase *θ_slope_* > 0	**81.22%**	**80.81%**	65.8%	62.7%
Significant increase *θ_slope_* > 0, *p* < 0.05	**22.91%**	**33.14%**	12.05%	9.32%
Extremely significant increase *θ_slope_* > 0, *p* < 0.01	9.47%	**20.03%**	4.83%	3.54%
Reduction *θ_slope_* < 0	18.78%	19.19%	**34.2%**	**37.3%**
Significant reduction *θ_slope_* < 0, *p* < 0.05	1.87%	**2.5%**	**2.65%**	1.78%
Extremely significant reduction *θ_slope_* < 0, *p* < 0.01	0.87%	**1.15%**	**0.91%**	0.4%

The bold font represents a larger proportion.

**Table 2:** The GRDs of NDVI in growing season to air temperature, precipitation, relative humidity and sunshine duration in February–June, March–July, April–August and May–September months from 2001 to 2017.

Climatic Factor	GRD			
	February–June	March–July	April–August	May–September
Air temperature	0.572	0.564	0.592	**0.613**
Precipitation	0.587	0.559	**0.616**	**0.616**
Relative humidity	0.610	**0.614**	0.610	0.610
Sunshine duration	**0.631**	0.613	0.622	0.620

The bold font represents a higher degree of correlation.

**Table 3:** The lag response of NDVI to Climate Factors in seven time periods.

Climatic Factor	Lag Response						
	2001–2011	2001–2012	2001–2013	2001–2014	2001–2015	2001–2016	2001–2017
Air temperature	Current month	Current month	Current month	Current month	Current month	Current month	Current month
Precipitation	Lag by 1 month	Lag by 1 month	Lag by 1 month	Current month	Current month	Lag by 1 month	Current month
Relative humidity	Lag by 2 months	Lag by 2 months	Lag by 2 months	Lag by 2 months	Lag by 2 months	Lag by 2 months	Lag by 2 months
Sunshine duration	Lag by 3 months	Lag by 3 months	Lag by 3 months	Lag by 3 months	Lag by 3 months	Lag by 3 months	Lag by 3 months
